# Applying Corporate Political Activity (CPA) analysis to Australian gambling industry submissions against regulation of television sports betting advertising

**DOI:** 10.1371/journal.pone.0205654

**Published:** 2018-10-16

**Authors:** Linda Hancock, Natalie Ralph, Florentine Petronella Martino

**Affiliations:** 1 Australian Research Council (ARC) Centre of Excellence for Electromaterials Science (ACES), Melbourne, Victoria, Australia; 2 Alfred Deakin Institute for Citizenship and Globalisation (ADICG), Deakin University, Melbourne, Victoria, Australia; 3 School of Psychology, Faculty of Health, Deakin University, Geelong, Australia; University of California San Francisco, UNITED STATES

## Abstract

**Aims:**

This research aimed to assess the application to the gambling industry, of Corporate Political Activity (CPA) analysis previously developed from public health research on tobacco industry interactions with political institutions and previously applied to the alcohol industry, but not the gambling industry.

**Background:**

A growing body of literature has confirmed how public interest outcomes are frequently opposed by vested interests. This research focused on gambling industry submissions to a 2013 Australian Parliamentary inquiry into sports betting advertising. Gambling advertising became highly controversial following deregulation of sports betting advertising in Australia subsequent to the 2008 Australian High Court Betfair challenge. The dramatic increase in gambling advertising during sporting event broadcasts at children’s viewing times and on new interactive technology, sparked public concerns. A series of national regulatory reviews followed and the gambling industry was actively involved in opposing further regulation.

**Method:**

The research used a corporate political activity (CPA) framework of analysis developed by UK tobacco public health researchers, which identified strategies and tactics used internationally by the tobacco industry, to broker pro-tobacco public policy outcomes. Testing the application of this CPA framework to gambling pro-industry strategies/tactics, this research focused on gambling industry submissions to the 2013 Australian Parliamentary Committee Inquiry.

**Results:**

Like the tobacco industry, the research found the gambling industry used identified strategies and tactics, some new tactics and a new strategy of ‘Corporate Social Responsibility’, promoting ‘responsible’ industry practices and pre-emptive establishment of internal ‘responsibility’ units/practices. Despite public concerns regarding sports betting advertising, the gambling industry reinforced individual choice/blame for harms and claimed it acted responsibly. It did this using strategies identified in the tobacco industry CPA framework: information strategy (and shaping the evidence base); financial incentive strategy; constituency building strategy; policy substitution strategy; legal strategy; and constituency fragmentation and destabilization strategy.

**Conclusion:**

Similar to the CPA analysis applied to tobacco and alcohol industries, the research demonstrated the usefulness of the CPA taxonomy for analyzing and documenting pre-emptive industry policy strategies and tactics, exposing gambling industry efforts to maintain industry self-regulation via voluntary codes and avoid more government regulation. Cross-sectoral application of the framework signals great potential for use of CPA by policymakers and public health advocates as a tool in the analysis of corporate industry arguments/discourses.

## Introduction

The influence of powerful vested interests over policy processes has posed barriers to the implementation of harm prevention public policies in industries known for potential harms. Corporate strategies to influence policies away from increased regulation have been well illustrated by the application of corporate political activity (CPA) analysis to tobacco industry (TI) influence [1] and alcohol industry influence against marketing regulation [2, 3], but not applied so far, to gambling.

This research focused on a regulatory controversy in Australia regarding conflicts over regulation of sports betting television advertising. Deregulation following the 2008 Australian High Court Betfair case (*Betfair Pty Limited v Western Australia*) [4] enabled a flood of gambling advertising during televised sports broadcasts including tennis, football, soccer, cricket and rugby national sports telecasts. This landmark national Australian High Court Betfair case overturned State/Territory restrictions on betting advertisements by interstate bookmakers. The judgement invalidated specific state-based restrictions on betting exchanges and resulted in removal of restrictions on advertising by wagering providers not licensed in that jurisdiction [4].The example was controversial because, not only was sports betting a new and exponentially expanding market for the gambling industry (GI), but due to sporting event exemptions to gambling advertising rules, this advertising occurred during family viewing times, newly exposing children to advertising of an adult-only (over 18) activity. At a time of declining participation in land-based gambling on electronic gambling machines and maturation of the racing wagering industry “limiting the prospects for further growth” [5], the issue of a newly expanding industry (even though it represents a small percentage of overall gambling revenue/participation), is a useful and relevant focus. In the context of public controversy surrounding a newly expanding industry, potential harm to minors and a national inquiry [5] with the potential to implement new regulations, industry submissions provided insight into their strident views aiming to influence government against further regulation.

In Australia, gambling on sport is a relatively new form of wagering compared with traditional and declining horse and dog racing. Sports betting is the only form of wagering to have increased over the last decade [6]. To put this in perspective, sports betting still represents only a small proportion of gambling revenue, which is still dominated by electronic gambling machines and casino gambling. But while electronic gambling machines account for much of gambling expenditure, average per capita gambling on machines, casinos and land-based venues whilst still high, has declined [7] and newer, more pervasive forms of gambling such as online sports betting have grown [7, 8, 9], with attendant harms [10]. Losses from sports betting increased by 30 percent from $626 million 2012/13 to $815 million in 2014/15 [7].

Sports betting expenditure has also increased at a faster rate than gambling in general for example, from 0.14 percent in 1994–95 to 3.8 percent in 2015–16 and the percentage change in sports betting turnover 2014–15 to 2015–16 was 35.1 percent compared to 5.5 percent for all gambling. [7]. It was on this basis that the Australian Productivity Commission recommended the Ministerial Council on Gambling review the 2010 television Code of Practice exemption relating to the promotion of lotteries, lotto, keno and sports betting during key children’s viewing periods (Recommendation 8.6) [11]. For the purposes of this analysis, testing the application of the taxonomy developed by tobacco researchers focused on a significant topical issue on which the GI mobilised its opposition to the potential threat of government interventions via detailed submissions.

By way of explanation to locate the research, gambling regulation in Australia is a mix of national and State/Territory responsibility. While the regulation and licensing of gambling (for example venue licensing, regulation of wagering including horse and greyhound racing, codes of practice and regulation of liquor licensed premises) are matters for State/Territory governments, issues falling under national regulation include broadcasting, consumer protection and online gambling. The Interactive Gambling Act 2001 sought to ban online gambling services (for example, poker and casino games) offered in Australia, with exemptions for wagering (including sports betting) via internet, telephone or digital television [12]. In terms of internet gambling, there is thus a complicated picture where some forms are permitted and others (casino and poker) are not. Similar to other jurisdictions, the borderless nature of the internet complicates implementation of the Interactive Gambling Act 2001 due to the accessibility of international online gambling providers and increases opportunities for gambling via internet enabled technologies both domestically and cross-border.

The research examined changing technology, gambling and debates on the impact of gambling advertising on youth, current regulation and failures to adapt regulation to the challenges posed by new betting technology platforms, such as the internet and smart phones. Testing the applicability of the CPA framework developed in 2014 by Savell, Gilmore and Fooks [1] for their analysis of TI influence over public policy, the research examined GI submissions to the 2013 Australian Parliamentary Joint Select Committee (2013) Inquiry into Advertising and Promotion of Gambling Services in Sport (2013 Select Committee Inquiry) and a proposed Bill on Broadcasting Services [5] This enabled examination of whether GI corporate interests adopted similar strategies and tactics to the TI, aimed at influencing public policy on GI marketing regulation. While the research could have focused on GI submissions to the 2010 Productivity Commission Inquiry [11], this was becoming dated and the submissions covered widely divergent issues. The main justification for focusing on the 2013 Select Committee Inquiry is that it was the culmination of a string of inquiries [13, 14] into a newly controversial gambling issue and it galvanised the sports betting industry and Clubs Australia (the peak body for registered and licensed clubs throughout Australia) to express their views about regulation. In the words of leading Australian gambling researchers, “(d)iscussion about the impact of the marketing of sports betting products has led to the most debate about gambling in Australia in recent years” [15].

The 2013 Select Committee Inquiry focused on the “pervasive nature of the promotion of sports betting, its integration into sporting commentary and the possible effects on children and vulnerable people from this high level of exposure” [5]. Public opinion was highly critical of the amount of advertising during sports broadcasts [5], and there were widespread public calls to end the exemption of sporting broadcasts from the ban on gambling advertising during sports broadcasts watched by children [16]. Notably, the reforms following the Inquiry (including the ban on live odds advertising) did not ban gambling advertising in general. (‘Live odds’ are the ‘real time’ bets that can be placed on specific parts of a game, and commentary teams often presented these during their commentary on a game.) This was despite research indicating the vulnerability of youth to gambling participation and problems, identifying gambling advertising as a risk factor for youth [8, 9, 17, 18], and despite widespread public concerns voiced during the inquiry [5]. The amended codes were favorable to industry, while failing to address issues of exposure of minors and community distain for sports betting advertising during event broadcasts [5].

## Gambling and youth’s vulnerability

Despite regulatory variations, there is widespread agreement on age limits to participation in gambling. In the United States it is 21 years of age, in the United Kingdom (UK), 18 (except for lotteries and arcade games), Australia 18, Canada 18 or 19 (depending on the province), and in Macau, people must be over 21 to gamble. Age-restrictions on potentially risky activities like gambling and alcohol, reflect the moral and ethical commitment of governments and regulators to the protection of minors as vulnerable persons and gambling and alcohol as adult-only activities. In the UK for example, this is expressed formally in the third licensing objective of the Gambling Act 2005, which specifies the importance of protecting children and other vulnerable persons “from being harmed or exploited by gambling” [19].

The Australian Productivity Commission [11] drew attention to the inconsistency between the general principles of restricting children’s exposure to gambling and the exemption granted to sporting event gambling advertising characterized by “continuously posted odds and the conspicuous identification of betting agencies”. In Australia, the overriding principle in general and in relation to youth, has been one of protection from harm on public health and consumer protection grounds [11], including concerns about the normalization of gambling (forming pro-gambling attitudes [20, 21]) for under-age minors.

The priority on prevention of exposure to gambling is informed by impact research that has shown the vulnerability of youth to gambling participation and subsequent problems [17, 22]. Crucially, research has identified gambling advertising as a risk factor for youth [8, 9, 17, 18], and adolescents as particularly attuned to gambling advertisements [23]. Children were found to be able to identify clearly gambling sponsorship aligned to Australia’s major sporting codes [24] and could name multiple sports betting brands [25].

## Failures to adapt regulation to changing technologies

Concerns about under age exposure to gambling have focused not only on advertising, but in combination with new gambling platforms, accessible through internet-connected devices widely used by young people and increasing concerns about harms associated with more pervasive forms of gambling like online sports betting [26, 27]. This facilitates marketing delivered by internet and during sporting events, at any time and any place. New of access via internet and internet-connected devices, such as smartphones and tablets, have smoothed the way for the expansion of internet-based sports gambling, which has become a thriving industry.

New online gambling markets have raised challenges for governments, the gambling industry and regulators. The European Parliament Resolution on the Integrity of Online Gambling recognized the need to prevent problem gambling and under-age gambling [28]. Compared with land-based gambling in venues, new online gambling technology is more accessible to under-age users and requires new regulatory thinking on how to regulate for probity, harm prevention and surveillance of participation by minors.

## Corporate Political Activity (CPA) analysis

CPA has been a focus of research on corporate influence since the 1980s, with CPA defined by Baysinger [29], as corporate attempts to shape government policy in ways favorable to the firm. Despite public health advocates’ efforts to bring research evidence on the harmful impacts of tobacco, alcohol and processed food to the notice of governments, it is only recently that the corporate political influence of the TI has undergone systematic analysis. Release of confidential TI documents has enabled analysis of corporate strategies applied by the TI to influence public health policy [30, 31, 32, 33, 34]. Recent CPA research, applied to ‘Big Tobacco’ [1], alcohol [2, 35] and (processed) food [35, 36], has demonstrated the effectiveness of CPA research in the analysis of industry tactics applied by, what might be considered, ‘dangerous consumption’ industries. However, CPA analysis has yet to be applied to the GI (although there have been some more narrowly focused studies of agenda setting and political ‘framing’) [37]. Common observations of gambling industry ‘wins’ on regulation have included watered down reforms, industry self-regulation via voluntary codes, covert concessions on rates and taxes, and government regulation that ‘turns a blind eye’ to industry misdeeds [11, 38, 39, 40]. The GI shares many similarities with ‘dangerous consumption’ industries, and the global explosion of gambling over the last two decades merits the inclusion of the GI in studies of “increased consumption of unhealthy commodities” [35]. CPA analysis is potentially useful to gambling industry researchers by identifying the strategies/tactics used by the GI.

## Method

### Procedure

The researchers accessed all 52 submissions [5] to the 2013 Australian 2013 Select Committee Inquiry. The 52 submissions were categorized in 2016/17 into eight stakeholder groups (as shown in S1 Table). Stakeholder groups included: 1) gambling/betting industry, including six submissions from GI companies and peak bodies; 2) broadcasting, including three peak bodies for television and radio, and telecommunications company, Telstra; 3) sport/racing industry including three peak bodies; 4) government/regulator including local, state and federal government bodies and gambling focused governance bodies; 5) non-governmental organizations (NGOs)/independent statutory bodies; 6) academic/research including health and gambling focused institutes and individual academics; 7) individuals; and 8) name withheld.

Gambling/betting industry submissions represented 11.53 per cent (6 out of 52) submissions to the 2013 Select Committee Inquiry. Inclusion criteria for analysis of submissions presented to the Inquiry, were that a submission be authored by a representative of a gambling/betting company or industry association. The six included submissions from Betfair Pty. Ltd. (Betfair); Sportsbet Pty. Ltd (Sportsbet); Tabcorp Holdings Ltd. (Tabcorp); Tom Waterhouse.com (Tom Waterhouse); Clubs Australia, the peak body for not-for-profit sports and community clubs; and Australian Wagering Council (AWC), the wagering peak body [5, 41, 42, 43, 44, 45]. AWC members include: Betfair; Sportsbet (including its subsidiary IASbet); Bet365; Betchoice (operating as Unibet); Eskander's Betstar; Sportingbet Group Australia (includes Sportingbet and Centrebet); and Tom Waterhouse. [5] With two peak bodies represented, the submissions arguably covered more providers than six, because peak bodies represented other wagering companies and community sector clubs across the country.

### Applying Savell et al.’s Corporate Political Activity taxonomy to GI industry submissions

Savell et al.’s [1] framework of CPA in the TI was adapted to analyze GI strategies/tactics aimed at achieving public policy outcomes. Building on Hillman and Hitt [46], Savell et al. [1] used ‘emergent coding’ to analyze 48 secondary source articles mentioning TI strategies/tactics and frames/arguments, where the industry had attempted to influence marketing regulation. They divided CPA into two taxonomies. The first taxonomy of ‘strategies’ incorporated ‘tactics’ (the methods used by a company to attempt to exert influence). They identified six strategies used by the TI: Information strategy; Financial incentive strategy; Constituency building strategy; Policy substitution strategy; Legal strategy; and Constituency fragmentation and destabilization strategy. They also developed a second taxonomy of ‘frames’, which incorporated ‘arguments’ (the reasons provided by a company for why they oppose or support an idea). The four frames included: Regulatory redundancy; Insufficient evidence; Negative Unintended Consequences; and Legal. Savell et al.’s CPA framework is shown in S2 Table.

The initial application of Savell et al.’s two discrete taxonomies in a pre-test across all six GI submissions, showed that all were to some extent applicable to the GI, but exposed significant overlap when attempting to separate ‘strategies/tactics’ from ‘frames/arguments’ by the two coders (LH and NR). For example, policy substitution under strategies/tactics overlapped with regulatory redundancy and the strategy of shaping the evidence overlapped with insufficient evidence. Similar issues were encountered by Martino, Miller, Coomber, Hancock and Kypri [2] in their application of Savell et al.’s framework to alcohol industry submissions to a 2013 Australian National Prevention Health Agency, in which case only one of the taxonomies (frames/arguments) was applied. Given the exploratory aims of this study, it was decided to examine in the first instance, the application of TI strategies/tactics to the GI so that this study showing how strategies/tactics were observed, was complementary to that of Martino et al. [2]. The application of the strategies/tactics framework combined both deductive and inductive approaches, with deductive thematic coding [47] of Savell et al.’s categories developed in relation to the TI tested for application to the GI. Following Martino et al.’s [2] application of CPA to alcohol submissions, a process of emergent inductive coding [48] was also used to identify new strategies/tactics that emerged as relevant to the GI, to be added to the framework.

The six GI submissions were coded independently using Savell et al.’s [1] strategies/tactics taxonomy by two researchers (LH and NR), with inter-coder discussions and cross-checking for amended coding. To strengthen intercoder reliability, decision rules for coding were developed for each strategy and tactic in the evolving framework, including new strategies and tactics. The two coders had in-depth discussions to arrive at consensus for coding purposes.

In terms of possible limitations to the research, the small number of submissions (six) is similar to the sample of eight used by Martino [2] when applying CPA to the alcohol industry and in any event, included two peak bodies with wider remit to land-based clubs [Clubs Australia] and to other sports betting organizations covered by the AWA. The Nvivo analysis undertaken may demonstrate selectivity by the coders (LH and NR) (although a coding frame and cross-checking process were implemented by using two analysts along with discussion of coding rules). Full use of the taxonomy may also need to use additional sources to submissions and industry reports; such as electoral donation registers. Further, the research relies on publicly available industry submissions which represent only part of the story, due to the lack of transparency in gambling and allied industry in-house research, and lobbying and formal and informal contact with government/politicians and policy developers, that is unavailable to the public.

## Results

The Australian GI used all six of Savell et al.’s [1] strategies to oppose national re-regulation of sports betting advertising during sporting event broadcasts in analysis of the six GI submissions. A new seventh strategy, ‘Corporate Social Responsibility’, and four new tactics were also identified. These are highlighted by shading in Table 1 below.

**Table 1:** Strategies/tactics observed in gambling industry submissions, using Savell et al.’s (2014) taxonomy. New strategies/tactics are highlighted.

Strategy	Tactic		Observed in gambling industry submissions (Yes/No)
1. Information	1a. Direct lobbying (meetings and correspondence with legislators/policymakers)		Y
	1b. Indirect lobbying (using third parties, including front groups, to lobby on the industry’s behalf)		Y
	1c. Shaping the evidence base	Commissioning, writing (or ghost writing), or disseminating research/publications	Y
		Preparing position papers, technical reports or data on impacts (including economic impact studies)	Y
	1d. Establishing industry/policymaker collaboration (e.g. via working group, technical group, advisory group)/work alongside policymakers providing technical support/advice		Y
	1e. Distorting the evidence		Y
	1f. Selectivity of evidence resulting in gaps and omissions in evidence		Y
2. Constituency building	2a. External constituency building	Form alliances with and mobilise other industry sectors/business/trade organisations	Y
		Media advocacy (press releases, publicity campaigns, public hearings, interviews)	Y
		Form alliances with or mobilise unions/civil society organisations/ consumers/employees/the public	Y
		Creation of front groups or astroturf organisations	Y (E.g. Clubs Australia)
	2b. Internal constituency building	Collaboration between companies/development of pan-industry group or industry trade association	Y (E.g. AWC)
3. Policy substitution	3a. Develop/promote (new or existing) voluntary code/self-regulation		Y
	3b. Develop/promote alternative regulatory policy		Y
	3c. Develop/promote non-regulatory initiative (generally seen to be ineffective/less effective, e.g. education programs)		Y
4. Legal	4a. Pre-emption		Y
	4b. Using litigation/threat of legal action		N (However, Betfair established success in litigation in the earlier 2008 Australian High Court Betfair case).
5. Constituency fragmentation and de-stabilisation	5a. Preventing the emergence of, neutralising and/or discrediting potential opponents (individuals, organisations or coalitions)		Y
6. Financial Incentive	6a. Providing current or offering future employment to those in influential role		Y (In terms of arguing how many jobs or amount of investment GI provides.)
	6b. Gifts, entertainment or other direct financial inducement		N*
7. Corporate Social Responsibility	7a. Industry commitment to ‘responsible’ operations		Y
	7b. Pre-emptive industry establishment of internal CSR units/practices, codes, sponsorship, training and volunteering		Y

Adapted from: Savell et al. [1].

* Inclusion of gifts and financial inducements is discussed later.

The results briefly discussed below, present examples from GI submissions that demonstrate the use of all strategies identified by Savell et al. [1] and the new strategy and tactics identified as relevant to GI CPA in our analysis, which need to be read in conjunction with S3 Table. (The numbers in text below refer to specific quotes and examples in S3 Table).

### Strategy 1. Information

The GI manages information via both direct and indirect lobbying tactics and by shaping the evidence base. Written submissions and giving verbal evidence to policy inquiries are a mechanism of direct lobbying. GI submissions/evidence analyzed here and submitted to the 2013 Select Committee Inquiry, are such examples. GI companies also made frequent cross-references to their submissions to other inquiries, as part of their direct lobbying (Betfair #1i).

Indirect lobbying involves using third parties including front groups, to lobby on the industry’s behalf. Companies and peak bodies in these relationships cited each other’s submissions and other research favorable to their position (Sportsbet #1ii). These bodies lobbied on a common platform of shared interests. The GI and associated media and sporting peak network partners included submissions to inquiries on their websites, as a means of disseminating their views.

The GI tactic of shaping the evidence base included disseminating research (Sportsbet #1iii) and preparing position papers or conducting research (Sportsbet #1iv), sometimes with partners. Betfair commissioned research on the impact of restrictions on online gambling in Germany by PriceWaterhouseCoopers, which argued that prohibitions on online gambling would result in a significant proportion of gamblers gravitating to unregulated sites [42]. The GI has established industry/policymaker collaborations; working cooperatively with government (Clubs Australia #1v).

Under the information strategy, submissions pointed to the need for two new tactics: ‘Distorting the evidence’ and ‘Selectivity of evidence resulting in gaps and omissions in evidence’. The GI distorted the evidence by arguing there is no evidence that inducements to customers impact on problem gambling (AWC #1vi), that most people are recreational non-problem gamblers (Tabcorp #1vii, Clubs Australia #1viii), that betting is secondary to consumers’ interests in sporting events (AWC #1ix), that online betting is not risky (AWC #1x), that the impact of gambling messages on children is unknown (AWC #1xi) and that only a small proportion of the sports viewing audience are children and that those who do, watch television with their parents. The GI selectively presented evidence (causing gaps and omissions), thereby making unsubstantiated claims. For example, arguing that most of those who bet on racing and sport online “do so safely” (AWC #1xii), overlooks recent evidence on harm from moderate and supposedly low risk gambling, and not just problem gambling [49]. Claims that “annual growth in wagering turnover has not significantly increased” (Sportsbet #1xiii) may be true for stagnating horse racing wagering but overlook evidence on the increased participation in sports betting [6, 20, 21]. Claims that problem gambling is lower for online wagering than land-based gambling (AWC #1xiv) overlooks recent findings of harms associated with online gambling [8, 10] alongside those already widely understood to be associated with electronic machine gambling in land-based venues. Sportsbet made assertions based on other research: “Free TV Australia in its submission to the inquiry also reports that of the very small percentage of the sports viewing audience comprised by children, more than 85 per cent co-view under adult supervision.” Sportsbet, 1xv]. This may be so, but does not detract from the evidence of negative impacts on children and the normalization of gambling [6, 20, 21], irrespective of whether or not they are viewing sports events with their parents.

### Strategy 2. Constituency building

Similar to the TI, the GI was engaged in constituency building with both external and internal collaborations. The GI made considerable effort via its peak bodies or front groups like AWC and Clubs Australia, to ally with other industry sectors such as sporting and/or broadcasting peak bodies. Tabcorp reflected on how “[p]rotecting the integrity of sports is a joint effort between government, sporting bodies and the wagering industry” (Tabcorp #2i). The GI collaborated with media and sports peak bodies, such as The Australian Subscription Television and Radio Association (ASTRA) and Coalition of Major Professional and Participation Sports (COMPPS) (Tabcorp #2iv, Clubs Australia #2v, Sportsbet #1ii). The GI used media advocacy including press releases and submissions (#2ii).

### Strategy 3. Policy substitution

All six submissions argue forcefully that rather than restrictions on industry gambling advertising, government should endorse industry self-regulation via industry codes and industry harm minimisation measures (AWC #3i, 3ii). The dominant message was that “industry Codes of Practice is a sufficient mechanism to protecting children from exposure to program material which may be harmful to them” (AWC #3iii) and that there was no need for government intervention (Betfair #3iv, Tabcorp #3v, Clubs Australia #3vi). The GI promoted alternative regulatory or legal measures to try to avoid tighter regulation in other more controversial policy areas (such as sports betting advertising). For example, the GI promoted alternative regulatory policy by supporting national voluntary standards for harm minimisation and consumer protection, but on the proviso, they are “achievable from an operational and technical perspective” (AWC #3vii). Further, the GI supported national legislation on sports integrity and the establishment of the National Integrity of Sport Unit and the National Policy on Match-Fixing in Sport (AWC #3viii, Tabcorp #3ix). The industry promoted non-regulatory initiatives like “responsible gambling advertising campaigns” and consumer education (Clubs Australia #3x), as policy substitution strategies.

### Strategy 4. Legal

Legal strategies include pre-emption and litigation/threat of legal action. Pre-emption was used as a legal term by Savell et al. [1] and identified in jurisdictional issues within the United States where federal law trumps conflicting state law. Australia has a Federal-State system based on thirty-nine Commonwealth heads of power (such as military defense, currency, quarantine and telecommunications described under Section 51 of the Australian Constitution) [50]. For the majority of policy domains, cooperative federalism prevails between the two levels, but on the proviso that States’ legislation will be deemed ineffective if inconsistent with or in a field covered by Commonwealth legislation (by virtue of the section 109 inconsistency provision) [50]. The Commonwealth collects income tax and the States annually broker deals on redistribution, with ongoing controversies over the redistribution of the goods and services tax. Recent High Court cases have determined that the heads of power of the Federal Parliament can be applied extensively, but in the gambling sphere this has not been tested, although Commonwealth powers for example under consumer protection, could give the Commonwealth jurisdiction. In Australian law, most gambling regulation prevails at State level (for example the license to operate), with the exception that telecommunications (including sports betting advertising) is a Commonwealth power. This explains why the GI is very active in putting their views to national Parliamentary inquiries into telecommunications law as a medium for gambling (or other) activities. In this respect, the GI was vocal in asserting domestic betting providers’ national “right to advertise” (AWC #4i, 4ii).

While the submissions to the 2013 Select Committee Inquiry did not reflect ‘litigation/threat of legal action’, Betfair had already established its success in litigation against government regulation in the 2008 Australian High Court Betfair case [4], which opened up the flood-gates to television gambling advertising during sporting events.

### Strategy 5: Constituency fragmentation and destabilisation

To destabilize critics’ advocacy linking TI and GI harms, the AWC argued that gambling is a popular form of entertainment that bears no similarity to the TI in terms of harm: (AWC #5v). In the GI submissions, with the participation of Clubs Australia representing land-based gambling interests, there was some evidence of industry demarcation and protection of their powerful land-based oligopoly from any increased regulation that could be metered out to telephone/online betting industry players. Indeed, Clubs Australia called for parity of regulation across land-based and online sectors, arguing that the status quo leaves online betting less regulated (Sportsbet #5i, Clubs Australia #5ii, 5iii, 5iv).

### Strategy 6. Financial incentive

Savell et al. [1] included two tactics under financial incentive: offering employment; and gifts, entertainment or other direct financial inducement. Their meta-analysis drew on research investigating corrupt TI activities. Corrupt GI activities are unlikely to be divulged in GI submissions to government inquiries, but these tactics have been kept as part of the taxonomy for applications where for example, electoral donations or corruption inquiries may find such incentives have been provided.

Submissions in the 2013 Select Committee Inquiry did however, argue how many jobs the GI has contributed to society: “Tabcorp is publicly listed on the Australian Securities Exchange and employs around 3,000 people across Australia” (Tabcorp #6i.).

### Strategy 7. Corporate social responsibility

A new strategy (with two new tactics 7a and 7b) was added to encapsulate GI promotion of Corporate Social Responsibility (CSR) commitment to ‘responsible gambling’ and operations, to cover where the GI pre-emptively establishes internal CSR (or similar) units/practices, sponsorship, training and volunteering. Industry claims to positive social contribution and responsibility represent attempts aimed at countering more substantive regulatory change. Under tactic 7a, industry argued its self-imposed ‘responsibility’ in relation to problem gambling, exotic betting, integrity risks (match fixing and organized crime) and drugs in sports (Tabcorp #7i, Sportsbet #7ii), and argued that its commitment to promotion of sports betting “in a socially responsible manner” (AWC #7iii). The industry stated that its responsible promotional activities avoid targeting children (AWC #7iv, Betfair #7v).

The new tactic, ‘Pre-emptive industry establishment of internal CSR units/practices, sponsorship, training and volunteering’, was illustrated in claims to sponsorship (AWC #7vi), setting up of internal integrity units (Tabcorp #7vii) and adopting national responsible gambling campaigns (Clubs Australia #7viii).

## Discussion

The application of Savell et al.’s [1] taxonomy, which was developed to analyze TI strategies/tactics used to oppose increased government regulation, certainly resonates with GI CPA. The results confirmed the strategies of (1) Information (and identified two additional tactics), (2) Constituency building, (3) Policy substitution, (4) Legal, (5) Constituency fragmentation and destabilisation, (6) Financial incentive, and reveals a new strategy of (7) Corporate Social Responsibility with 2 new tactics.

Governments and the GI have principally framed their approaches to gambling regulation in terms of a government-endorsed industry commitment to ‘responsibility’ via voluntary industry codes of practice, where their agreed construction of responsible gambling was framed in terms of the *individual’s* responsibility to avoid harm and to act responsibly [38, 39]. The results discussed above and detailed in S3 Appendix, are discussed below, and demonstrate that the GI used the same strategies as the TI to ward off government regulatory reform and to protect their vested interests in maintaining the status quo, in this case, industry self-monitoring of sports betting advertising during sporting event broadcasting. Perhaps reflecting more strict regulation of the TI, such as laws banning advertising tobacco products, TI arguments emphasized the financial and legal impacts of increased regulation [1]. Alternatively, the GI basically argued increased regulation is not necessary. Similar to the GI, the alcohol industry has also argued it is sufficiently responsible under current self-regulation [2, 51].

### Information

Analysis of the GI found strong evidence in submissions for all four of the Information tactics identified by Savell et al. [1] in relation to the TI, and two additional tactics: ‘Distorting the evidence’ and ‘Selectivity of evidence resulting in gaps and omissions in evidence’. While it could be argued that these might have been subsumed under ‘shaping the evidence base,’ this did not fit with Savell et al.’s criteria under that header. The coders did not think this was sufficiently detailed to differentiate out more extreme or stronger cases, where the GI distorted the evidence (and there is research evidence to the contrary) and where GI selectivity in evidence presentation resulted in gaps or omissions. It was considered useful to draw out more extreme instances than may be implied by ‘shaping the evidence base,’ which under Savell et al. referred to writing papers and position papers, whereas these new examples refer to the GI putting forward certain views on what constitutes ‘evidence’.

Corporate lobbying (both direct and indirect) is a well-identified tool used to influence political decision-makers [11, 52]. In public policy deliberations, information asymmetry exists between the GI, government and community. The GI has inside information from its own research and data. Gambling researchers in the UK have found evidence where the GI distorts the evidence in its public policy advocacy and makes false claims [53, 54]. Researchers on public policy framing and vested interests in addictions research have found how alcohol industry public relations information strategies have sought to frame (and limit) the range of issues under consideration [55, 56]. Given the focus of public debates and the 2013 Select Committee Inquiry on harm to children from sports betting advertising, the gambling and allied industries sought to frame their desired policy outcomes (self-regulation and business as usual) as a compromise, focused on gambling as an individual social problem. This deflected attention from the issue of public distain of sports betting advertising and broader negative impacts like the normalization of gambling among minors and vulnerable groups [25, 26].

Previous research studies show that the gambling and allied industries seek to shape the evidence base and distort the evidence via their submissions to inquiries, evidence-giving and in-house commissioned research that is not subject to peer review, un-coincidentally, with outcomes favorable to industry [53, 54]. To shape the evidence base, and arguably, distort it, television industry peak bodies, FreeTV and ASTRA gave evidence to the 2013 Select Committee Inquiry, asserting the low percentage of children watching sports programs relative to the total viewing audience; although information regarding their research and its peer review, methodology and assessment was not divulged [57, 58]. It can also be argued that these percentages still represent significant numbers of children; a point that was ignored during evidence giving.

In terms of establishing industry/policymaker collaborations (such as through providing technical support and advice), the submissions did not explicitly give examples of this. They did however, emphasize the GI’s focus on continuing to work cooperatively with government [43] and argued for instance, that “[p]rotecting the integrity of sports is a joint effort between government, sporting bodies and the wagering industry” [45]. Co-regulatory models fostered by collaborations between industry and government can position industry as a partner/collaborator rather than a provider of risky products, and frame policy issues in terms of balance and compromise favoring industry interests; rather than a primary focus on public health, harm prevention, or consumer interests. For example, “The club’s industry has worked cooperatively with state and territory governments for many years to implement proven, cost effective harm minimization policies” [43]. Further, the National Rugby League (NRL) stated: “We do work very hard to internally regulate, and we do very often work with government to try and make sure that we are heading in the right direction” [59].

Although not evident from submissions, vested interests also donate to political parties [60, 61]. Other research has found that gambling providers are linked to political parties via donations, with the GI declaring A$1,294,501 in donations (an under-estimate) to Australian political parties in 2015–16. The biggest donors included the Australian Hotels Association ($522,478) and Clubs NSW ($155,603) [61].

### Constituency building

Constituency building is a foundation of corporate political strategy [62]. The growth in sports betting has resulted in an increase in the advertising of sports betting products and services. It has also broadened the pro in-game advertising lobbying constituency to include the GI and its peak bodies, broadcasting networks, sports venues and advertising agencies in a mutually beneficial business network oriented to market expansion. Reinforcing this view, Livingstone and Woolley [63] describe cricket and football commentators as ''embedded marketing agents'' for big online betting companies. This highlights how Savell et al.’s [1] ‘external’ and ‘internal’ constituency building tactics almost merge for the GI, as the GI and allied industries have become tightly woven in a broader industry chain of supply, with shared interests. Shared interests link sports gambling and the broader GI to hotels, clubs, and casinos, and with the betting agency TAB, broadcasters, advertisers, and sports clubs like football and rugby that are reliant on gambling funding for player purchases and club expenses.

### Policy substitution

Savell et al. [1] found strong global evidence for TI use of policy substitution, and in particular voluntary industry self-regulation, as a key strategy aimed at preventing legislated marketing regulation that may risk bans. In terms of policy substitution benefiting GI interests, the 2013 Select Committee Inquiry referred to a ‘co-regulatory’ framework where “(t)he codes of practice form a co-regulatory framework that broadcasters operate under” [5]. This diverted attention away from banning sports betting advertising altogether during broadcasts by focusing on company voluntary self-regulation rather than prohibition. Substituting what look like feasible strategies diverted attention from harsher outcomes.

Concurrently, the GI extolled the virtues of consumer choice and free markets over regulation to promote industry self-regulation rather than more invasive mandatory government regulations. Policy substitution tactics also included a focus on common agreement on issues peripheral to the issue at hand. In this case, the consideration of a ban on advertising during sporting events was eclipsed by industry pre-emptive voluntary actions such as ‘responsible gambling’ codes and agreeing to industry codes that stop live odds during sports event broadcasts. This strategy diverted debate away from more punitive approaches such as implementing a total ban on gambling advertisements, despite public opinion for re-reregulation from prominent researchers. “Researchers and politicians have increasingly called for the closure of a loophole that allows sports betting advertisements during televised sporting events or current affairs programs prior to the 8:30 pm watershed (the time after which adult content may be broadcast)” [64].

The GI submissions reflected an additional pre-emptive diversionary tactic. This was illustrated by GI support for national legislation to regulate match fixing and other sports integrity issues. Backing this up, the early establishment of integrity units by the Australian Football League and NRL were promoted as proactive support for integrity in sport [5]. These organizations also promoted their internal player and staff education programs on problem gambling, organized crime and corruption [65]. Such issues while important, are peripheral to whether there should be a blanket ban on gambling advertising during sporting events, but they served to shore-up an industry-government co-regulatory approach for business as usual.

### Legal

Savell et al. [1] referred to this strategy as “using the legal system” and identified two tactics used by the TI: preemption and legal action. They argued threatening legal action has been commonly used globally by the TI, especially against packaging regulation. In the case of tobacco, the World Health Organization Framework Convention on Tobacco Control recognized that a comprehensive ban on advertising, promotion and sponsorship would reduce the consumption of tobacco products [66].

In the absence of a parallel convention linking gambling promotion, increased frequency of gambling and harms requiring ratifying nations to legally ban gambling advertising, examples of this strategy were slightly different to those outlined by Savell et al. GI submissions preemptively asserted a competitive free market and the GI ‘right to advertise’. Gambling was thus normalized, as gambling advertising is embedded in sports entertainment in the context of the free market. There are parallels with TI tactics, where research demonstrating damage from active-smoking and the inhalation of second-hand smoke is largely negated by cigarette advertising which communicates the opposite, especially to young people, that smoking is a harmless, healthy and socially enhancing behavior [67]. A similar preemptive message is communicated for gambling; that gambling (and advertising) is fundamental to the leisure of watching sport [23]. Promoting consumer choice, the GI has systematically supported publicly-funded ‘problem gambling’ programs, also reinforcing an individual choice/blame model for problem gambling, rather than a consumer protection, harm prevention perspective that would lead to bans on gambling advertising [39, 40, 53].

Of note, legal action supported by well-resourced GI multinational corporations has been used to win national regulatory victories, such as the 2008 Australian High Court case brought by Betfair [4] to stop State/Territories legislating against their marketing. This victory opened new markets for the industry in the subsequently deregulated environment, resulting in opportunistic gambling advertising. As an alternative to more costly and time-consuming legislation (and potential industry legal action), industry codes and self-regulation have been favored by both government and the industry [38]. These are presented as a fair balance between the wellbeing of the community, the regulatory burden and financial implications imposed on industry. Industry codes and self-regulation are regularly used as a tactic to broker compromises that can undermine public health policy outcomes [38, 39, 53, 54].

### Constituency fragmentation and destabilisation

Savell et al. [1] referred to this as a strategy aimed at “weakening opponents”. This strategy aims to split allegiances and broker complex compromises. Consequently, policy outcomes tend not to address the public interest issues at hand, and deliver a compromise supporting industry interests. For example, in GI arguments, the protection of youth (by for example, a ban on all gambling advertising) was played off against market-based interests (revenue), resulting in fragmented and inconsistent policy outcomes. Further, government has been complicit in calling for more research; thus, delaying action on policy reform. Echoing GI assertions, the Australian Prime Minister Gillard and Minister Conroy argued at a press conference in May 2013 [68], they supported a ban on live odds and restrictions on gambling advertisements during sporting matches but could not take deeper action regarding a ban on gambling advertising, until further research and accurate information regarding the effects of the advertisements on children was gathered [69].

Representing terrestrial-based operators at the time of the inquiry, the Clubs Australia peak body played an interesting role differentiating its own commitments to ‘responsible gambling’ and arguing that online/telephone gambling providers are under-regulated. Any new industry-wide regulations could unsettle Clubs Australia’s constituents and perhaps undermine their profit. Until recently, Clubs Australia’s members were in open competition with the online sector (but have since sought to partner with an operator in order to gain a higher share of wagering revenue). During the Inquiry, Clubs Australia played a boundary maintaining role, seeking to demarcate terrestrial clubs from online providers and thereby protect its own interests in a powerful national oligopoly [43].

### Financial incentive

Financial incentive tactics discovered by investigative research by Savell et al. were not identified in the GI submissions as the GI is unlikely to voluntarily disclose the use of financial incentives, such as gifts or political donations. Financial incentive tactics could be broadened in the case of gambling to include financial incentives such as taxation, sponsorships, and advertising revenue. These pose conflict of interest issues for governments and allied industries, such as sports venues and broadcasters [69, 70]. Such financial incentive tactics are shaped by shared interests between the GI, government and allied industries. Gambling taxes are a lucrative on-going source of State/Territory government revenue [11] and decreased gambling, for example brought about by a blanket ban on television gambling advertising, would reduce State/Territory taxes. It was also estimated at the time, that a crackdown on live odds betting would decrease gambling revenue for metropolitan television networks from $40 million to $30 million [69], but was less damaging to industry interests than a total ban.

Profit-sharing/commissions between the GI, sports clubs and broadcasters constitute a form of financial partnership that underpins the combined front of gambling and allied industries in a mutually beneficial chain of supply. For example, advertising revenue from the live broadcast of sports events has been a significant revenue stream for commercial and subscription television broadcasters and media interests [70]. Hence, profit-sharing contributes to the united front opposing a ban on sports betting advertising during sports event broadcasts.

### Corporate social responsibility

Although not formally part of the Savell et al. framework, research on the TI has identified CSR as a reputation management tool and a method of securing access to policymakers [71]. Interestingly, Martino et al. also added it to their study of alcohol industry submissions against regulation [2]. TI political philanthropy has been used for constituency building, third party advocacy, weakening opposing political constituencies, facilitating access to or political leverage of policymakers, enhancing corporate image and credibility, implementing political CSR and influencing the tobacco control agenda [71, 72, 73,74]. Examples in political science illustrate how CSR is used for lobbying government and corporate reputation management via partnerships with respected NGOs and other organizations [75, 76]. The GI submissions currently analyzed, mentioned sponsorship of various sporting teams, sporting codes and sporting facilities as benevolent industry contributions to supporting sport [77]. While these could have been coded under strategy 3 as an example of developing or promoting a non-regulatory initiative, it is more powerful to differentiate out initiatives that industry is likely to identify as CSR. The CSR literature has also established how strategic CSR ties corporate giving directly to motivations of corporate reputation and influence-enhancement [78]. Similarly, research applying CPA to the alcohol industry found that claims to corporate responsibility such as membership of DrinkWise, a front body for the industry, was used by the alcohol industry to create a credible image aimed at influencing policy advisors/governments to delay increased marketing regulation [2].

Promoting consumer choice within the frame of ‘responsible gambling’, the GI has systematically supported publicly-funded ‘problem gambling’ programs, reinforcing individual choice/blame for problem gambling rather than a consumer protection, harm prevention perspective [39, 53, 54]. For the GI, CSR focuses on voluntary industry codes for responsible gambling, support for sports betting related integrity issues, and support for problem gambling counselling services [79].

## Conclusion

The 2013 Select Committee Inquiry outcome ultimately recommended the status quo while recommending the current exemption of gambling advertising for sporting programs be reviewed; stating that, “(t)he changes do not affect general gambling advertising which would continue to be allowed during a sporting match” [5].

Sports-betting is a rapidly growing market in Australia and internationally [11]. Sports betting corporate players–often in coalitions of interest with broader GI corporates, broadcasters, advertisers and sports venues–are powerful, well-resourced proponents of self-interested influence on public policy. Sports betting advertising is a relatively new national political and social issue in Australia, that emerged in the wake of the 2008 Australian High Court Betfair case challenge to State/Territory betting advertising regulation. Although industry codes prohibit most gambling advertisements in children’s viewing times, sports events and programs have been exempt from this code under provisions originally designed to protect traditional wagering. With multiple platforms (including internet and social media), expanding into gambling and used by youth and adults alike, public policy needs to keep pace with regulation that is adequate to the task of harm prevention and consumer protection, especially concerning new media.

The aim of the current research was to ascertain the usefulness of Savell et al.’s [1] CPA framework, particularly the strategies/tactics taxonomy, for application to GI submissions to the Australian Parliamentary 2013 Select Committee Inquiry into Advertising and Promotion of Gambling Services in Sport. This study indicated that CPA is a useful approach for systematically analyzing GI strategies/tactics and attempts to influence public policy on sports betting regulation. The framework’s power lies in the systematic nature of the typology focus on industry strategies and tactics and identification of a range of strategies/tactics, rather than isolated examples of single strategies or tactics. This research contributes to a newly emerging area of enquiry in addiction industry studies “as a recognized field of enquiry” and “a necessary companion to other forms of public health research because, without it, public health recommendations will continue to be ignored” [80].

In the future, CPA analysis can be applied to other gambling public policy issues and used to monitor the industry’s political activities. Using an iterative approach, application to other GI policy activity will potentially enable further development of CPA as a useful analytical tool. By exposing the shared platform across problematic consumption industries (tobacco, alcohol, processed food and gambling), CPA analysis is useful as an evolving policymaker and activist framework, for its focus on generic corporate strategies/tactics. This signals great potential for the analysis of corporate discourses, power and interests, irrespective of sector. The next step would be to attempt to combine the two Savell et al. [1] taxonomies of strategies/tactics, and frames/arguments, that have been applied separately to both alcohol [2] and gambling industries.

## Supporting information

S1 Table Submissions to 2013 Australian Parliamentary Joint Select Committee by stakeholder groups. (DOCX)

S2 Table Savell et al. (2014) Strategies and Tactics used by the Tobacco Industry when Attempting to Influence Marketing Regulation. (DOCX)

S3 Table Examples of strategies/tactics observed in gambling industry submissions, applying Savell, Gilmore and Fooks’ 2014 taxonomy, and illustrating new strategies/tactics identified for the gambling industry. (DOCX)

S1 Appendix Submissions to the 2013 Australian Parliamentary Joint Select Committee by stakeholder groups. (DOCX)

S2 Appendix Framework developed by Savell et al. in 2014. (DOCX)

S3 Appendix Examples of strategies/tactics observed in gambling industry submissions, applying Savell et al.’s 2014 taxonomy, and illustrating new strategies/tactics identified for the gambling industry. (DOCX)
