# Supplementary material for: Applying Corporate Political Activity (CPA) analysis to Australian gambling industry submissions against regulation of television sports betting advertising
Source: PLoS One. 2018 Oct 16;13(10):e0205654. doi: 10.1371/journal.pone.0205654 (PMC6191115; doi:10.1371/journal.pone.0205654)
Supplement: S1 Table — (DOCX) [file pone.0205654.s001.docx]

Supplementary Table I: Submissions to 2013 Australian Parliamentary Joint Select Committee by stakeholder groups.

| **Stakeholder Group** | **N** | **Contributors (Referred to in this paper)** |
| --- | --- | --- |
| Gambling industry (included in analysis) | 6 | - Two main wagering operators:  Tabcorp  Betfair Pty. Ltd. (internet and telephone betting)    -Sportsbet Pty. Ltd  -Tom Waterhouse.com (bookmaker)  - Australian Wagering Council (AWC), the wagering peak body ^[[1]](#footnote-1)^  -Clubs Australia |
| Broadcasting | 4 | -FreeTV (Peak body for commercial television)  -Commercial Radio Australia  -Telstra Broadcast Services  -ASTRA Subscription Television Australia |
| Sport/racing industry | 3 | -The Coalition of Major Professional and Participation Sports (COMPPS) (Peak body)  - Australian Racing Board (peak body for [Thoroughbred](https://en.wikipedia.org/wiki/Thoroughbred) racing)  - Harness Racing Australia |
| Government/regulator | 5 | -Victorian Local Governance Association  -Tasmanian Gaming Commission  -Victorian Responsible Gambling Foundation  - Office for Sport, Department of Regional Australia, Local Government, Arts and Sport  - Australian Crime Commission |
| NGO/independent statutory body | 5 | -Family Voice Australia  -Relationships Australia  -The Australian Psychological Society  -Royal Australian and New Zealand College of Psychiatrists  - Gambling Impact Society |
| Academic/University/research | 5 | -University of Sydney Gambling Treatment Clinic  -Health Promotion Evaluation Unit, University of Western Australia  -Communications Law Centre, University of Technology Sydney  -Associate Professor Peter Katelaris  -Associate Professor Samantha Thomas |
| Individual | 17 | Mr Peter Mair, Mr Bill Ranken, Mr Kevin Dennehy, Ms Judith Cox, Mr Daniel Odell, Mr Jim Russell, Mr John Nolan, Mr Paul McCabe, Mr Christopher Dodd, Mr Max Vardanega, Mr Trevor Rowe, Dr Greg Tanner, Ms Colleen Pont, Mr Jim Carlton, Mr Michael Cuddihy, Mr David Shliahov, Dr Bruce Blunden |
| Name withheld | 7 | Name withheld |
| Total | 52 |  |

1. The peak industry body for the online wagering and sportsbetting industry. Its members are: Betfair; Bet365; Betchoice (operating as Unibet); Eskander's Betstar; Sportsbet (including its subsidiary IASbet); Sportingbet Group Australia (which includes Sportingbet and Centrebet); and Tom Waterhouse.com. See Australian Wagering Council, Submission 31, p. 1. [↑](#footnote-ref-1)
