# Supplementary material for: Applying Corporate Political Activity (CPA) analysis to Australian gambling industry submissions against regulation of television sports betting advertising
Source: PLoS One. 2018 Oct 16;13(10):e0205654. doi: 10.1371/journal.pone.0205654 (PMC6191115; doi:10.1371/journal.pone.0205654)
Supplement: S2 Table — (DOCX) [file pone.0205654.s002.docx]

**Supporting Information**

**Table S2 Savell et al. (2014) Strategies and Tactics used by the Tobacco Industry when Attempting to Influence Marketing Regulation**

| **Strategy** | **Tactic** | |
| --- | --- | --- |
| Information | Direct lobbying (meetings and correspondence with legislators/policymakers) | |
|  | Indirect lobbying (using third parties, including front groups, to lobby on the industry’s behalf) | |
|  | Shaping the evidence base | Commissioning, writing (or ghost writing), or disseminating research/publications |
|  |  | Preparing position papers, technical reports or data on impacts  (including economic impact studies) |
|  | Establishing industry/policymaker collaboration (e.g. via working group, technical group, advisory group)/work alongside policymakers providing technical support/advice | |
| Constituency building | External constituency building | Form alliances with and mobilise other industry sectors/business/trade organisations |
|  |  | Media advocacy (press releases, publicity campaigns, public hearings, interviews) |
|  |  | Form alliances with or mobilize unions/civil society organizations/ consumers/employees/the public |
|  |  | Creation of front groups or astroturf organisations |
|  | Internal constituency building | Collaboration between companies/development of pan-industry group or industry trade association |
| Policy substitution | Develop/promote (new or existing) voluntary code/self-regulation | |
|  | Develop/promote alternative regulatory policy | |
|  | Develop/promote non-regulatory initiative (generally seen to be ineffective/less effective, e.g. education programmes) | |
| Legal | Pre-emption | |
|  | Using litigation/threat of legal action | |
| Constituency fragmentation  and destabilization | Preventing the emergence of, neutralising and/or discrediting potential opponents (individuals, organisations or coalitions) | |
| Financial Incentive | Providing current or offering future employment to those in influential role | |
|  | Gifts, entertainment or other direct financial inducement | |

**Source: Savell E, Gilmore AB, Fooks G (2014) How Does the Tobacco Industry Attempt to Influence Marketing Regulations? A Systematic Review. PLoS ONE 9(2): e87389. Table 2.**

**Table S2 Arguments used by the Tobacco Industry when attempting to influence marketing regulation.**

| **Frame** | **Sub-frames (where applicable)** | | **Argument** |
| --- | --- | --- | --- |
| Negative Unintended Consequences | Economic | Manufacturer | The cost of compliance for manufacturers will be high/the time required for implementation has been underestimated |
|  |  |  | Regulation will result in financial or job losses (among manufacturers) |
|  |  |  | The regulation is discriminatory/regulation will not affect all producers/customers equally |
|  |  | Public Revenue | Regulation will cause economic/financial problems (for city, state, country or economic area (e.g. European Union)) |
|  |  | Associated Industries | Regulation will result in financial or job losses (among retailers and other associated industries, e.g. printing, advertising, leisure) |
|  | Public Health |  | Regulation will have negative public health consequences |
|  | Illicit Trade |  | Regulation will cause an increase in illicit trade |
|  | Other |  | Regulation could have other negative unintended consequences (e.g. cause confusion amongst customers, set a precedent for other types of products/’slippery slope’) |
| Legal |  |  | Infringes legal rights of company (trademarks, intellectual property, constitutionally protected free speech (e.g. US First Amendment), international trade agreements) |
|  |  |  | Regulation is more extensive than necessary/regulation is disproportionate |
|  |  |  | Body doesn’t have the power to regulate/it’s beyond their jurisdiction |
|  |  |  | Regulation will cause an increase in compensation claims |
| Regulatory Redundancy |  |  | Industry adheres to own self-regulation codes/self-regulation is working well |
|  |  |  | Industry only markets to those of legal age/is actively opposed to minors using product |
|  |  |  | Existing regulation is satisfactory/existing regulation is satisfactory, but requires better enforcement |
| Insufficient Evidence |  |  | There’s insufficient evidence that the proposed policy will work / marketing doesn’t cause or change behavior (it’s only used for brand selection and capturing market share), so regulation will have no effect |
|  |  |  | The health impacts of consumption remain unproven |

**Source: Savell E, Gilmore AB, Fooks G (2014) How Does the Tobacco Industry Attempt to Influence Marketing Regulations? A Systematic Review. PLoS ONE 9(2): e87389. Table 3.**
