# Supplementary material for: Applying Corporate Political Activity (CPA) analysis to Australian gambling industry submissions against regulation of television sports betting advertising
Source: PLoS One. 2018 Oct 16;13(10):e0205654. doi: 10.1371/journal.pone.0205654 (PMC6191115; doi:10.1371/journal.pone.0205654)
Supplement: S3 Table — (DOCX) [file pone.0205654.s003.docx]

**Appendix S3: Examples of strategies/tactics observed in gambling industry submissions, applying Savell, Gilmore and Fooks’ 2014 taxonomy, and illustrating new strategies/tactics identified for the gambling industry**

| **Gambling industry strategies/tactics**  **(Savell, Gilmore and Fooks, 2014)**  ***New strategies/tactics in italics*** | | **Example/quote from gambling industry submissions** | **Assigned Example/**  **Quote number** |
| --- | --- | --- | --- |
| **Strategy 1. Information**  **(**used in all 6 gambling industry submissions)  1a. Direct lobbying | | Illustrated by the gambling industry’s (GI) submissions to inquiries.  “Betfair welcomes the opportunity to make a submission to the Joint Select Committee’s (Committee) inquiry into the advertising and promotion of gambling services in sport (Inquiry).  Betfair has previously addressed the vast majority of the terms of reference of this Inquiry in its previous submissions to the Committee and its two submissions to the Department of Broadband, Communications and the Digital Economy’s (Department) review of the Interactive Gambling Act 2001 (Cth) (IGA Review).” [Betfair] | 1i |
| 1b. Indirect lobbying | | Gambling corporates’ indirect lobbying via membership of peak bodies, or coalitions with peak bodies in broadcasting (ASTRA, FREETV) and sports bodies (Coalition of Major Professional and Participation Sports (COMPPS)) due to commercial profit-sharing relationships, which cite each other’s submissions.  For example, Sportsbet cites research reported by The Australian Subscription Television and Radio Association (ASTRA) about the small proportion of children watching live sports events. | 1ii |
| 1c. Shaping the evidence base | Commissioning, writing (or ghost writing), or disseminating research/publications | Sportsbet [60] states, “On 5 November 2012, the AWC lodged a submission with the South Australian Independent Gambling Authority in response to the Authority’s review of its existing Codes of Practice, including an Issues Paper published by the Authority titled ‘Issues Paper 3 – Inducements’. We have attached the AWC’s submission, and summarised the central issues”. [Sportsbet] | 1iii |
|  | Preparing position papers, technical reports or data on impacts  (including economic impact studies) | “The Committee found in its Second Report in December 2011 that the effects, if any, on children viewing gambling messages during sports broadcasts are unknown. There remains a dearth of research on this issue and further research in this area is needed to underpin effective policy making. Sportsbet welcomes the call for research by Gambling Research Australia (GRA) on the marketing of sports-betting and racing, as well as the use of social media in gambling.  Relevantly, the AWC has recently committed to working on a research project by GRA to investigate the use of social media by wagering operators together with the use of responsible gambling messaging. It is anticipated that each of these areas of research will provide comprehensive data upon which to base policies which cover young people who are increasingly using emerging technologies in the new media environment.” [Sportsbet] | 1iv |
| 1d. Establishing industry/policymaker collaboration | | For example, Clubs Australia and the AWC.  “The clubs industry has worked cooperatively with state and territory governments for many years to implement proven, cost effective harm minimisation policies” [Clubs Australia]. | 1v |
| 1e. ***New Tactic:*** ***Distorting the evidence*** | | “There is no evidence that indicates the availability of inducements to customers encourages or has increased the prevalence of problem gambling” [AWC].  “The vast majority of our customers are recreational punters who, on average, place bets of $13.” [Tabcorp]  “While most people can enjoy gambling responsibly, a small minority of people having difficulties in controlling their gambling expenditure; as a result their excessive gambling causes harm, for themselves and for their families that warrants a targeted harm minimisation approach.” [Clubs Australia]  “Betting on sports is generally considered a secondary reason for most consumers to follow a sporting event and the Productivity Commission (2010) inquiry found that only a relatively small share of the $19 billion total gambling spend in Australia was attributed to sportsbetting which was estimated at just 1.2% or $200 million.” [AWC]  “The AWC rejects the argument that online wagering and sportsbetting is a more risky environment for those who are at risk of developing problem gambling behaviours. Rather the AWC believes that the online environment, provided that the operators are licensed and regulated, affords enhanced protection to customers and provides responsible operators with vital insight into any potential issues.” [AWC]  AWC states “it is noted in the Joint Select Committee’s own report’s (December 2011) findings that the effects, if any, on children viewing gambling messages during sports broadcasts are unknown.” [AWC] | 1vi  1vii  1viii  1ix  1x  lxi |
| 1f. ***New Tactic: Selectivity of evidence resulting in gaps and omissions in evidence*** | | “The AWC also rejects as grossly misleading the comparisons, which have been drawn between the regulated online sports betting and wagering industry and tobacco products, in an attempt to ban sports betting advertising. There is no safe level of tobacco use. By contrast, the overwhelming majority of the 2 million Australians who bet on racing and sport online do so safely. What’s more, it is a legitimate form of entertainment for them.” [AWC response to questions]  “Despite a public perception that there has been an ‘explosion’ in wagering turnover which, in part, has been driven by the lifting of the prohibition (in 2008) on corporate bookmakers advertising, overall annual growth in wagering turnover has not significantly increased. Annual growth in wagering turnover is typically 4-5 per cent per annum.”  [SportsBet]  “The incidence of problem gambling is reportedly significantly lower in online wagering in comparison to land-based gaming with the Productivity Commission reporting that it was estimated that 80-85 per cent of problem gambling was directly related to the use of poker machines.” [AWC]  Sportsbet [60] states, “at least two submissions to this inquiry highlight the general misconception regarding the numbers of children actually watching live sports broadcasts. The Australian Subscription Television and Radio Association (ASTRA) reports that persons under 18 comprise a very small proportion of the audience for live sports events on subscription TV8. Free TV Australia in its submission to the inquiry also reports that of the very small percentage of the sports viewing audience comprised by children, more than 85 per cent co-view under adult supervision.” [Sportsbet] | 1xii  1xiii  1xiv  1xv |
| **Strategy 2. Constituency building**  **(**used in 5 GI submissions)  2a. External constituency building | Form alliances with and mobilise other industry sectors/business/trade organisations | “Protecting the integrity of sports is a joint effort between government, sporting bodies and the wagering industry. Tabcorp is committed to continuing to work with government and sporting bodies to achieve this objective.” [Tabcorp] | 2i |
|  | Media advocacy (press releases, publicity campaigns, public hearings, interviews) | The 2013 Select Committee Inquiry analysed in this paper is an example of a ‘public hearing’. All submitters make active use of media releases on their submissions. | 2ii |
|  | Form alliances with or mobilise unions/civil society organisations/ consumers/employees/the public | For example, Tabcorp 2013 integrity agreement with International Sports Monitoring GmbH in respect of betting on the 2012 London Olympics. [Tabcorp] | 2iii |
|  | Creation of front groups or astroturf organisations | For example, the AWC and Clubs Australia. |  |
| 2b. Internal constituency building | Collaboration between companies/development of pan-industry group or industry trade association | For example, the GI peak bodies AWC and Clubs Australia. Further, in the broader industry chain of supply, representing shared interests across the GI, venues, sports and broadcasting entities, there is significant collaboration. Tabcorp [50] highlights its:  “Liaison with sporting bodies and other stakeholders   - Working closely with sports controlling bodies and complying with requests not to offer bets on certain bet types, including certain ‘spot’ markets. - Engagement with COMPPS (Coalition of Major Professional and Participation Sports) including regular reporting to it on Tabcorp's integrity program.”   “Clubs Australia submits that a collaborative approach between local sporting codes and sports- betting providers is the best approach to tackling issues associated with the integrity of sport. They should work together constructively and transparently, to seek efficient and effective means for protecting the integrity of sport in Australia.” [Clubs Australia] | 2iv  2v |
| **Strategy 3. Policy substitution**  **(**used in 6 GI submissions)  3a. Develop/promote (new or existing) voluntary code/self-regulation | | “The AWC is working with the various advertising standards bodies to adopt codes to standardize the regulations around the promotion of live odds.” [AWC]  “the AWC suggests that a better approach would be a requirement that, in circumstances where advertising is reaching a broad audience, it be a condition that wagering operators employ appropriate harm minimisation measures. This would encourage all operators to provide services in a responsible manner.” [AWC]  “The AWC also believes that the Broadcasting Services Act 1992 (Cth) (BSA) which provides for public policy concerns to be addressed through industry Codes of Practice is a sufficient mechanism to protecting children from exposure to program material which may be harmful to them.” [AWC]  “Betfair is of the view that the most appropriate way to regulate sports betting advertising is through a nationally consistent code-of-conduct applying to all sports and racing advertising.” [Betfair]  “Tabcorp has robust measures in place that govern its own advertising practices.” [Tabcorp]  “governments must resist the temptation to adopt emotionally-driven, reactive solutions and provide sufficient time for the sporting codes to digest the contents of ACC report and respond through the implementation of their own self-regulatory measures.” [Clubs Australia] | 3i  3ii  3iii  3iv  3v  3vi |
| 3b. Develop/promote alternative regulatory or legal policy  (*Diversionary focus on GI support for national legislation (e.g. on sports integrity) to pre-empt tighter regulation in controversial policy areas)* | | “The AWC supports the development of national standards for harm minimisation and consumer protection that covers responsible gambling, advertising, licensing requirements and probity standards, provided they are evidence based, not detrimental to the competitiveness of the industry and reasonably achievable from an operational and technical perspective.” [AWC]  “The AWC welcomed the establishment of the National Integrity of Sport Unit (NISU)15 and  more recently the AWC publicly called on all remaining States and Territories to join Victoria, NSW and South Australia in introducing consistent national criminal offences for those found guilty of corrupting the betting outcomes of sporting events.” [AWC]  “Tabcorp supports the efforts of Australian governments to manage sports integrity and welcomes the National Policy on Match-Fixing in Sport which was signed by Australia’s Sports Ministers on 10 June 2011. We also support the introduction of nationally consistent criminal penalties for people who corrupt sporting events and the establishment of the National Integrity of Sport Unit.” [Tabcorp] | 3vii  3viii  3ix |
| 3c. Develop/promote non-regulatory initiative | | “Clubs Australia believes all gambling providers should be encouraged to undertake industry funded responsible gambling advertising campaigns. Socially responsible advertising campaigns like Clubs Australia’s Part of the Solution campaign help to promote a culture of responsible gambling….. Clubs Australia believes a key to success in preventing future instances of unsafe online sports- betting among youth is the adoption of an educative approach.” [Clubs Australia] | 3x |
| **Strategy 4. Legal**  (used in 3 GI submissions)  4a. Pre-emption | | The GI argue that federal rights (e.g. to advertise) should prevail. This sets out to undermine stricter State/Territory initiatives on advertising. For example, “The right to advertise is an important distinguishing legal right granted only to those operators who have agreed to meet Australian standards and are subject to the strict Australian regulatory environment.” [AWC]  “it is an offence for offshore wagering and sportsbetting operators to advertise their services in Australia. This right to advertise is an important distinguishing legal right granted only to those operators, such as AWC members, who have agreed to meet Australian standards and are subject to the strict Australian regulatory environment.” [AWC] | 4i  4ii |
| 4b. Using litigation/threat of legal action | | Prior to the inquiry, Betfair established its success litigating against government regulation in the 2008 High Court of Australia Betfair case. |  |
| **Strategy 5: Constituency fragmentation and destabilisation**  **(**used in 3 GI submissions)  5a. Preventing the emergence of, neutralising and/or discrediting potential opponents (individuals, organisations or coalitions) | | There was some evidence of different parts of the GI arguing to discredit each other. For example, ‘wedge politics’ between online sports betting and terrestrial gambling interests (e.g. Sportsbet, a telephone/online betting firm and Clubs Australia, the peak body for terrestrial venue-based gambling).  “The existing prohibition to offer inducements to open a betting account (e.g. Vic, NSW) and/or to bet more frequently on an existing account (e.g. NSW, WA) favours retail-based operators (TABs) and distorts a competitive wagering market” [SportsBet]  “In our view, sports-betting, whether conducted online or at land-based venue, is a legitimate recreational activity enjoyed by many Australians. However, Clubs Australia is concerned that overly pervasive gambling advertising coupled with inappropriate advertising practices, such as offering inducements to gamble and promoting credit betting, could potentially lead to an exacerbation of problem gambling in Australia.” [Clubs Australia]  “The online gambling environment poses a number of additional risks to consumers in comparison to traditional terrestrial forms of gambling including:  - convenient 24 hour access on any internet enabled computer or mobile device - ability to play in private and without supervision - ability to gamble using credit - difficulties in preventing access by minors - gambling using credit facilities.” [Clubs Australia]  “A number of online sites aggressively promote credit card gambling by offering free bets and other sign-up bonuses in exchange for credit card details….. Research indicates online gamblers are three to four times more likely to develop gambling problems compared to those people who gamble with more traditional land-based gambling services1. Despite the obvious additional risks posed by online sports-betting few regulations exist governing the responsible conduct of online gambling.” [Clubs Australia]  “The AWC also rejects as grossly misleading the comparisons, which have been drawn between the regulated online sports betting and wagering industry and tobacco products, in an attempt to ban sports betting advertising. There is no safe level of tobacco use. By contrast, the overwhelming majority of the 2 million Australians who bet on racing and sport online do so safely. What’s more, it is a legitimate form of entertainment for them.” [AWC questions on notice] | 5i  5ii  5iii  5iv  5v |
| **Strategy 6. Financial Incentive**  **(**used in 2 GI submissions)  6a. Providing current or offering future employment to those in influential role | | Identified in terms of arguing how many jobs the GI provides and what it contributes as an industry. Otherwise, it is difficult to ascertain this from inquiry submissions.  “Tabcorp is publicly listed on the Australian Securities Exchange and employs around 3,000 people across Australia” [50] | 6i |
| 6b. Gifts, entertainment or other direct financial inducement | | Unlikely disclosure in submissions* |  |
| **Strategy 7.**  ***New Strategy: Corporate Social Responsibility (used in 6 GI submissions)***  ***7a. New tactic: Industry commitment to ‘responsible’ operations*** | | “Tabcorp has continually led the industry in the development and introduction of responsible gambling initiatives which is why we have been recognised as global leader in the promotion of responsible gambling by the Dow Jones Sustainability Index.” [Tabcorp]  “Sportsbet is strongly committed to providing wagering services in a responsible manner so as to minimise the incidence of problem gambling in the community and consistent with this, Sportsbet has in place a wide range of responsible gambling measures.” [SportsBet]  “The AWC is committed to ensuring the advertising and promotion of sports betting by its members is undertaken in a socially responsible manner and accords with the promotion of responsible gambling and the need to ensure the protection of the integrity of sport.” [AWC]  “AWC members do not directly target their industry advertising and promotional strategies to children.” [AWC]  “Betfair believes that it is important that the advertising and promotion of sports betting is conducted in a responsible manner and importantly should never encourage gambling by those that are underage.” [Betfair] | 7i  7ii  7iii  7iv  7v |
| ***7b. New Tactic: Pre-emptive industry establishment of internal CSR units/practices, sponsorship, training and volunteering.*** | | “AWC members make a significant investment in the Australian economy and into Australian racing and sports through sponsorship of various sporting teams, sporting codes and sporting facilities.” [AWC]  “An Integrity business unit has been established within the company’s Corporate, Legal and Regulatory division. The Integrity unit has responsibility for working with sports controlling bodies, regulators and police on integrity matters which involves regular contact with these stakeholders.” [Tabcorp]  “The Australian Hospitality and Leisure Group have an award winning responsible gambling campaign with former AFL star David Swartz as its ambassador. Clubs Australia has a national responsible gambling campaign entitled ‘Part of the Solution’ which informs club patrons of the various support measures provided by clubs to assist them should they start to develop a problem with their gambling such as access to free 24-hour counselling services and self exclusion schemes.” [Clubs Australia] | 7vi  7vii  7viii |

Adapted from Savell, Gilmore, Fooks [1:4].

* 6b is retained for application of the taxonomy to broader sources such as political donations.
