# Supplementary material for: Applying Corporate Political Activity (CPA) analysis to Australian gambling industry submissions against regulation of television sports betting advertising
Source: PLoS One. 2018 Oct 16;13(10):e0205654. doi: 10.1371/journal.pone.0205654 (PMC6191115; doi:10.1371/journal.pone.0205654)
Supplement: S1 Appendix — (DOCX) [file pone.0205654.s004.docx]

**Appendix S1: Submissions to the 2013 Australian Parliamentary Joint Select Committee by stakeholder groups**

| **Stakeholder Group** | **N** | **Contributors** |
| --- | --- | --- |
| 1.Gambling/betting industry (included in analysis) | 6 | - Tabcorp  - Betfair  - Sportsbet  - Tom Waterhouse  - Australian Wagering Council (AWC)  - Clubs Australia-Tom Waterhouse.com |
| 2.Broadcasting | 4 | - FreeTV  - Commercial Radio Australia  - Telstra Broadcast Services  - ASTRA Subscription Television Australia |
| 3.Sport/racing industry | 3 | - The Coalition of Major Professional and Participation Sports (COMPPS)  -Australian Racing Board  -Harness Racing Australia |
| 4.Government/regulator | 5 | - Victorian Local Governance Association  - Tasmanian Gaming Commission  - Victorian Responsible Gambling Foundation  - Office for Sport, Department of Regional Australia, Local Government, Arts and Sport  - Australian Crime Commission |
| 5.NGOs/independent statutory bodies | 5 | - Family Voice Australia  - Relationships Australia  - The Australian Psychological Society  - Royal Australian and New Zealand College of Psychiatrists  - Gambling Impact Society |
| 6.Academic/University/  research | 5 | - University of Sydney Gambling Treatment Clinic  - Health Promotion Evaluation Unit, University of Western Australia  - Communications Law Centre, University of Technology Sydney  - Associate Professor Peter Katelaris  - Associate Professor Samantha Thomas |
| 7.Individual | 17 | Mr Peter Mair, Mr Bill Ranken, Mr Kevin Dennehy, Ms Judith Cox, Mr Daniel Odell, Mr Jim Russell, Mr John Nolan, Mr Paul McCabe, Mr Christopher Dodd, Mr Max Vardanega, Mr Trevor Rowe, Dr Greg Tanner, Ms Colleen Pont, Mr Jim Carlton, Mr Michael Cuddihy, Mr David Shliahov, Dr Bruce Blunden |
| 8.Name withheld | 7 | Name withheld |
| Total | 52 |  |
